# 6-Chloro-2-methyl-4-phenyl-3-[1-phenyl-5-(2-thien­yl)-4,5-dihydro-1*H*-pyrazol-3-yl]quinoline

**DOI:** 10.1107/S1600536809040239

**Published:** 2009-10-10

**Authors:** Hoong-Kun Fun, Ching Kheng Quah, S. Sarveswari, V. Vijayakumar, R. Prasath

**Affiliations:** a X-ray Crystallography Unit, School of Physics, Universiti Sains Malaysia, 11800 USM, Penang, Malaysia; b Organic Chemistry Division, School of Science and Humanities, VIT University, Vellore 632 014, India

## Abstract

In the title mol­ecule, C_29_H_22_ClN_3_S, the quinoline ring system, thio­phene ring and phenyl ring substituents are inclined at angles of 71.70 (7), 59.26 (9) and 81.61 (9)°, respectively, to the 4,5-dihydro­pyrazole ring. In the 4-phenyl­quinoline ring system, the phenyl ring makes a dihedral angle of 62.49 (7)° with mean plane of quinoline ring system. In the crystal structure, mol­ecules are linked *via* weak inter­molecular C—H⋯N hydrogen bonds, forming an extended one-dimensional chain along the *b* axis and are further consolidated by C—H⋯π and π–π stacking inter­actions [centroid–centroid distances = 3.7022 (10) Å].

## Related literature

For general background to quinolines and their derivatives, see: Morimoto *et al.* (1991 ▶); Michael (1997 ▶); Markees *et al.* (1970 ▶); Campbell *et al.* (1988 ▶). For applications of quinolines, see: Maguire *et al.* (1994 ▶); Kalluraya & Sreenivasa (1998 ▶); Roma *et al.* (2000 ▶); Chen *et al.* (2001 ▶); Skraup (1880 ▶). For the synthesis of new quinoline derivatives, see: Katritzky & Arend (1998 ▶); Jiang & Si (2002 ▶). For related structures, see: Fun *et al.* (2009*a* ▶,*b* ▶). For the stability of the temperature controller used for the data collection, see: Cosier & Glazer (1986 ▶).
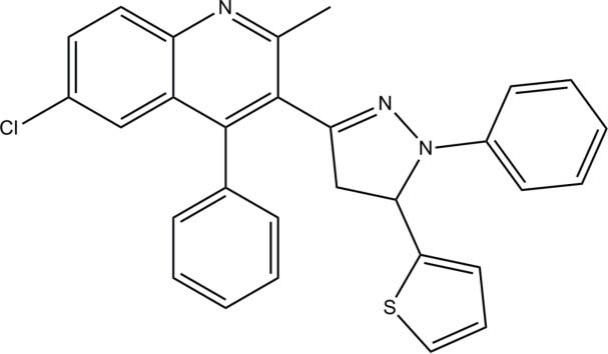


## Experimental

### 

#### Crystal data

C_29_H_22_ClN_3_S
*M* *_r_* = 480.01
Monoclinic, 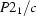

*a* = 14.0395 (4) Å
*b* = 9.4199 (3) Å
*c* = 19.3020 (6) Å
β = 114.696 (2)°
*V* = 2319.22 (12) Å^3^
*Z* = 4
Mo *K*α radiation
μ = 0.28 mm^−1^
*T* = 100 K
0.54 × 0.51 × 0.21 mm

#### Data collection

Bruker SMART APEXII CCD area-detector diffractometer
Absorption correction: multi-scan (**SADABS**; Bruker, 2005 ▶) *T*_min_ = 0.863, *T*_max_ = 0.943
32081 measured reflections
6723 independent reflections
5814 reflections with *I* > 2σ(*I*)
*R*_int_ = 0.051

#### Refinement

*R*[*F* ^2^ > 2σ(*F* ^2^)] = 0.052
*wR*(*F* ^2^) = 0.139
*S* = 1.07
6723 reflections
308 parameters
H-atom parameters constrained
Δρ_max_ = 1.19 e Å^−3^
Δρ_min_ = −0.39 e Å^−3^

### 

Data collection: *APEX2* (Bruker, 2005 ▶); cell refinement: *SAINT* (Bruker, 2005 ▶); data reduction: *SAINT*; program(s) used to solve structure: *SHELXTL* (Sheldrick, 2008 ▶); program(s) used to refine structure: *SHELXTL*; molecular graphics: *SHELXTL*; software used to prepare material for publication: *SHELXTL* and *PLATON* (Spek, 2009 ▶).

## Supplementary Material

Crystal structure: contains datablocks global, I. DOI: 10.1107/S1600536809040239/lh2922sup1.cif

Structure factors: contains datablocks I. DOI: 10.1107/S1600536809040239/lh2922Isup2.hkl

Additional supplementary materials:  crystallographic information; 3D view; checkCIF report

## Figures and Tables

**Table 1:** Hydrogen-bond geometry (Å, °)

*D*—H⋯*A*	*D*—H	H⋯*A*	*D*⋯*A*	*D*—H⋯*A*
C15—H15*A*⋯N1^i^	0.93	2.60	3.490 (2)	161
C3—H3*A*⋯*Cg*1^ii^	0.93	2.63	3.481 (2)	152
C12—H12*A*⋯*Cg*1^iii^	0.93	2.83	3.487 (2)	129
C17—H17*B*⋯*Cg*2	0.97	2.88	3.6916 (19)	142
C20—H20*A*⋯*Cg*3^iii^	0.93	2.89	3.7523 (18)	155
C21—H21*A*⋯*Cg*4^iv^	0.93	2.84	3.6084 (18)	141
C22—H22*A*⋯*Cg*3^v^	0.93	2.88	3.5750 (18)	132
C29—H29*B*⋯*Cg*5^vi^	0.96	2.89	3.694 (2)	142

Symmetry codes: (i) 
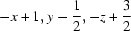
; (ii) 
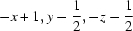
; (iii) 
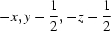
; (iv) 
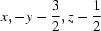
; (v) 
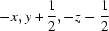
; (vi) 
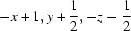
. *Cg*1–*Cg*5 are centroids of the S1/C19–C22, C10–C15, C23–C28, N1/C1/C6–C9 and C1–C6 rings, respectively.

**Fig. 1.**
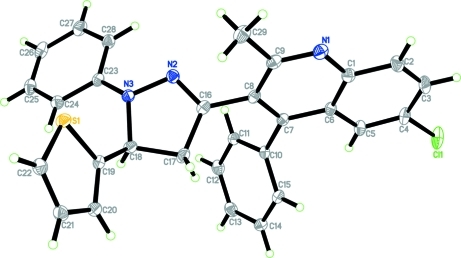
The molecular structure of the title compound with atom labels and 50% probability ellipsoids for non-H atoms.

**Fig. 2.**
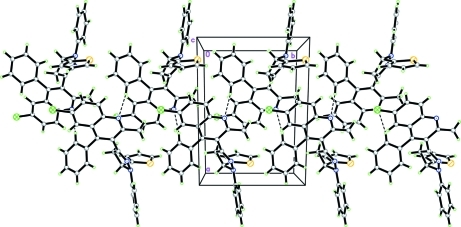
The crystal packing of title compound, viewed along the c axis. Intermolecular hydrogen bonds are shown as dashed lines.

## supplementary crystallographic information

### Comment

Quinolines and their derivatives are very important compounds because of their wide occurrence in natural products (Morimoto *et al.*, 1991; Michael, 1997) and biologically active compounds (Markees *et al.*, 1970; Campbell *et al.*, 1988). A large variety of quinolines have interesting physiological activities and found attractive applications as pharmaceuticals, agrochemicals and as synthetic building blocks (Maguire *et al.*, 1994; Kalluraya & Sreenivasa, 1998; Roma *et al.*, 2000; Chen *et al.*, 2001; Skraup, 1880). Many synthetic methods such as Skraup, Doebner-Von Miller, Friedländer and Combes reactions have been developed for the preparation of quinolines. Due to their great importance, the synthesis of new derivatives of quinoline remains an active research area (Katritzky & Arend, 1998; Jiang & Si, 2002).

The title molecule (Fig. 1) consists of a 4-phenylquinoline ring system (N1/C1–C15), a thiophene ring (S1/C19–C22) and a phenyl ring (C23–C28) attached to a 4,5-dihydropyrazole ring (N2/N3/C16–C18). The 4,5-dihydropyrazole ring is inclined at angles of 71.70 (7), 59.26 (9) and 81.61 (9)° with respect to the quinoline group, thiophene and phenyl rings substituted to 4,5-dihydropyrazole ring, respectively. In the 4-phenylquinoline ring system, the substituent phenyl ring (C10–C15) forms a dihedral angle of 62.49 (7)° with mean plane of quinoline ring system (N1/C1–C9). Bond lengths and angles are within normal ranges, and comparable to closely related structures (Fun *et al.*, 2009*a*,*b*).

In the crystal structure (Fig. 2), the molecules are linked *via* weak intermolecular C—H···N hydrogen bonds to form an extended one-dimensional chain along the *b*-axis and are further consolidated by C–H···π (Table 1) and π–π stacking interactions between S1/C19–C22 (centroid *Cg*1) and N2/N3/C16–C18 (centroid *Cg*2) rings, with a *Cg*1···*Cg*2 distance of 3.7022 (10) Å.

### Experimental

A mixture of 1-(6-chloro-2-methyl-4-phenylquinolin-3-yl)-3-(thiophen-2-yl) prop-2-en-1-one (0.4 g 0.001 *M*) and phenyl hydrazine (0.756 g 0.007 *M*) in distilled ethanol was refluxed for about 8 h. The resulting mixture was concentrated to remove the ethanol and then poured onto ice and neutralized with dilute HCl. The resultant solid was filtered, dried and purified by column chromatography using 1:1 mixture of chloroform and petroleum ether. *M.p.* 463-465K.

### Refinement

All H atoms were positioned geometrically and refined using a riding model, with C—H = 0.93–0.98 Å and *U*_iso_(H) = 1.2 or 1.5 *U*_eq_(C). A rotating-group model was applied for the methyl group.

### Crystal data

**Table:**

C_29_H_22_ClN_3_S	*F*(000) = 1000
*M**_r_* = 480.01	*D*_x_ = 1.375 Mg m^−^^3^
Monoclinic, *P*2_1_/*c*	Mo *K*α radiation, λ = 0.71073 Å
Hall symbol: -P 2ybc	Cell parameters from 9914 reflections
*a* = 14.0395 (4) Å	θ = 2.7–35.6°
*b* = 9.4199 (3) Å	µ = 0.28 mm^−^^1^
*c* = 19.3020 (6) Å	*T* = 100 K
β = 114.696 (2)°	Block, yellow
*V* = 2319.22 (12) Å^3^	0.54 × 0.51 × 0.21 mm
*Z* = 4	

### Data collection

**Table:**

Bruker SMART APEXII CCD area-detector diffractometer	6723 independent reflections
Radiation source: fine-focus sealed tube	5814 reflections with *I* > 2σ(*I*)
graphite	*R*_int_ = 0.051
φ and ω scans	θ_max_ = 30.0°, θ_min_ = 2.2°
Absorption correction: multi-scan (*SADABS*; Bruker, 2005)	*h* = −19→19
*T*_min_ = 0.863, *T*_max_ = 0.943	*k* = −13→13
32081 measured reflections	*l* = −27→27

### Refinement

**Table:**

Refinement on *F*^2^	Primary atom site location: structure-invariant direct methods
Least-squares matrix: full	Secondary atom site location: difference Fourier map
*R*[*F*^2^ > 2σ(*F*^2^)] = 0.052	Hydrogen site location: inferred from neighbouring sites
*wR*(*F*^2^) = 0.139	H-atom parameters constrained
*S* = 1.07	*w* = 1/[σ^2^(*F*_o_^2^) + (0.0722*P*)^2^ + 1.7861*P*] where *P* = (*F*_o_^2^ + 2*F*_c_^2^)/3
6723 reflections	(Δ/σ)_max_ = 0.001
308 parameters	Δρ_max_ = 1.19 e Å^−^^3^
0 restraints	Δρ_min_ = −0.39 e Å^−^^3^

### Special details

**Table:**

Experimental. The crystal was placed in the cold stream of an Oxford Cyrosystems Cobra open-flow nitrogen cryostat (Cosier & Glazer, 1986) operating at 100.0 (1) K.
Geometry. All e.s.d.'s (except the e.s.d. in the dihedral angle between two l.s. planes) are estimated using the full covariance matrix. The cell e.s.d.'s are taken into account individually in the estimation of e.s.d.'s in distances, angles and torsion angles; correlations between e.s.d.'s in cell parameters are only used when they are defined by crystal symmetry. An approximate (isotropic) treatment of cell e.s.d.'s is used for estimating e.s.d.'s involving l.s. planes.
Refinement. Refinement of *F*^2^ against ALL reflections. The weighted *R*-factor *wR* and goodness of fit *S* are based on *F*^2^, conventional *R*-factors *R* are based on *F*, with *F* set to zero for negative *F*^2^. The threshold expression of *F*^2^ > σ(*F*^2^) is used only for calculating *R*-factors(gt) *etc*. and is not relevant to the choice of reflections for refinement. *R*-factors based on *F*^2^ are statistically about twice as large as those based on *F*, and *R*- factors based on ALL data will be even larger.

### Fractional atomic coordinates and isotropic or equivalent isotropic displacement parameters (Å^2^)

**Table:**

	*x*	*y*	*z*	*U*_iso_*/*U*_eq_	
Cl1	0.52535 (3)	0.15347 (5)	0.55993 (3)	0.02753 (12)	
S1	0.13358 (3)	0.98946 (4)	0.82776 (2)	0.01644 (10)	
N1	0.46753 (10)	0.73714 (15)	0.64499 (8)	0.0179 (3)	
N2	0.16075 (10)	0.79726 (15)	0.65110 (8)	0.0152 (3)	
N3	0.09408 (10)	0.81949 (15)	0.68774 (7)	0.0142 (3)	
C1	0.47887 (12)	0.59890 (18)	0.62770 (9)	0.0161 (3)	
C2	0.56285 (12)	0.5667 (2)	0.60768 (10)	0.0199 (3)	
H2A	0.6082	0.6385	0.6075	0.024*	
C3	0.57797 (12)	0.4312 (2)	0.58854 (10)	0.0211 (3)	
H3A	0.6338	0.4105	0.5762	0.025*	
C4	0.50776 (12)	0.32362 (19)	0.58782 (10)	0.0190 (3)	
C5	0.42605 (12)	0.34935 (18)	0.60750 (9)	0.0167 (3)	
H5A	0.3812	0.2762	0.6069	0.020*	
C6	0.41072 (11)	0.48866 (17)	0.62882 (9)	0.0143 (3)	
C7	0.32784 (11)	0.52504 (17)	0.65019 (8)	0.0131 (3)	
C8	0.31640 (11)	0.66558 (17)	0.66620 (9)	0.0142 (3)	
C9	0.38941 (12)	0.76979 (17)	0.66316 (9)	0.0163 (3)	
C10	0.25384 (11)	0.41394 (16)	0.65240 (9)	0.0130 (3)	
C11	0.14714 (12)	0.42336 (17)	0.60459 (9)	0.0155 (3)	
H11A	0.1223	0.4986	0.5705	0.019*	
C12	0.07765 (12)	0.32076 (18)	0.60763 (10)	0.0189 (3)	
H12A	0.0067	0.3276	0.5755	0.023*	
C13	0.11390 (13)	0.20823 (18)	0.65841 (10)	0.0200 (3)	
H13A	0.0674	0.1396	0.6603	0.024*	
C14	0.22015 (13)	0.19852 (18)	0.70645 (10)	0.0198 (3)	
H14A	0.2446	0.1234	0.7407	0.024*	
C15	0.28996 (12)	0.30060 (18)	0.70350 (9)	0.0175 (3)	
H15A	0.3609	0.2934	0.7357	0.021*	
C16	0.23190 (12)	0.70685 (17)	0.68943 (9)	0.0148 (3)	
C17	0.22254 (14)	0.65035 (19)	0.75936 (10)	0.0215 (3)	
H17A	0.2833	0.6753	0.8055	0.026*	
H17B	0.2139	0.5480	0.7569	0.026*	
C18	0.12318 (12)	0.72575 (17)	0.75567 (9)	0.0146 (3)	
H18A	0.0676	0.6558	0.7467	0.018*	
C19	0.14343 (11)	0.80776 (16)	0.82741 (9)	0.0131 (3)	
C20	0.17644 (12)	0.75300 (18)	0.89937 (9)	0.0165 (3)	
H20A	0.1862	0.6566	0.9105	0.020*	
C21	0.19417 (13)	0.86128 (18)	0.95550 (9)	0.0176 (3)	
H21A	0.2174	0.8429	1.0073	0.021*	
C22	0.17337 (13)	0.99422 (18)	0.92477 (9)	0.0172 (3)	
H22A	0.1799	1.0768	0.9528	0.021*	
C23	−0.01218 (11)	0.84260 (16)	0.63906 (9)	0.0136 (3)	
C24	−0.08776 (12)	0.83902 (17)	0.66885 (9)	0.0156 (3)	
H24A	−0.0676	0.8222	0.7205	0.019*	
C25	−0.19302 (12)	0.86073 (17)	0.62083 (10)	0.0186 (3)	
H25A	−0.2429	0.8563	0.6407	0.022*	
C26	−0.22460 (12)	0.88873 (19)	0.54400 (10)	0.0203 (3)	
H26A	−0.2950	0.9032	0.5124	0.024*	
C27	−0.14923 (13)	0.89488 (19)	0.51478 (10)	0.0205 (3)	
H27A	−0.1697	0.9144	0.4633	0.025*	
C28	−0.04358 (12)	0.87221 (18)	0.56158 (9)	0.0174 (3)	
H28A	0.0060	0.8768	0.5414	0.021*	
C29	0.38103 (13)	0.92253 (18)	0.68234 (11)	0.0221 (3)	
H29A	0.4414	0.9737	0.6847	0.033*	
H29B	0.3774	0.9280	0.7308	0.033*	
H29C	0.3189	0.9635	0.6438	0.033*	

### Atomic displacement parameters (Å^2^)

**Table:**

	*U*^11^	*U*^22^	*U*^33^	*U*^12^	*U*^13^	*U*^23^
Cl1	0.01621 (19)	0.0284 (2)	0.0367 (3)	0.00178 (15)	0.00981 (17)	−0.01431 (19)
S1	0.02017 (19)	0.01186 (18)	0.01700 (19)	0.00090 (13)	0.00746 (15)	0.00049 (14)
N1	0.0130 (6)	0.0176 (7)	0.0196 (7)	−0.0012 (5)	0.0033 (5)	0.0006 (5)
N2	0.0131 (6)	0.0157 (6)	0.0170 (6)	−0.0001 (5)	0.0065 (5)	0.0000 (5)
N3	0.0127 (6)	0.0169 (6)	0.0120 (6)	0.0025 (5)	0.0042 (5)	0.0021 (5)
C1	0.0114 (6)	0.0185 (7)	0.0154 (7)	−0.0003 (5)	0.0026 (5)	0.0007 (6)
C2	0.0119 (6)	0.0259 (8)	0.0208 (8)	−0.0020 (6)	0.0058 (6)	0.0007 (7)
C3	0.0112 (6)	0.0297 (9)	0.0219 (8)	0.0007 (6)	0.0066 (6)	−0.0023 (7)
C4	0.0125 (7)	0.0225 (8)	0.0202 (8)	0.0029 (6)	0.0049 (6)	−0.0045 (6)
C5	0.0122 (6)	0.0178 (7)	0.0190 (7)	0.0003 (5)	0.0054 (6)	−0.0032 (6)
C6	0.0101 (6)	0.0163 (7)	0.0150 (7)	0.0003 (5)	0.0035 (5)	−0.0002 (5)
C7	0.0099 (6)	0.0149 (7)	0.0121 (6)	0.0004 (5)	0.0022 (5)	0.0002 (5)
C8	0.0117 (6)	0.0149 (7)	0.0131 (7)	0.0019 (5)	0.0023 (5)	0.0003 (5)
C9	0.0143 (6)	0.0153 (7)	0.0150 (7)	−0.0001 (5)	0.0018 (5)	0.0003 (6)
C10	0.0121 (6)	0.0133 (7)	0.0143 (6)	0.0005 (5)	0.0062 (5)	−0.0013 (5)
C11	0.0126 (6)	0.0168 (7)	0.0169 (7)	0.0012 (5)	0.0061 (5)	0.0016 (6)
C12	0.0137 (7)	0.0212 (8)	0.0222 (8)	−0.0030 (6)	0.0079 (6)	−0.0025 (6)
C13	0.0216 (7)	0.0173 (8)	0.0261 (8)	−0.0039 (6)	0.0147 (7)	−0.0030 (7)
C14	0.0238 (8)	0.0153 (7)	0.0240 (8)	0.0038 (6)	0.0136 (7)	0.0046 (6)
C15	0.0153 (7)	0.0169 (7)	0.0202 (8)	0.0044 (5)	0.0074 (6)	0.0027 (6)
C16	0.0148 (6)	0.0132 (7)	0.0140 (7)	0.0007 (5)	0.0036 (5)	−0.0018 (5)
C17	0.0269 (8)	0.0224 (8)	0.0159 (7)	0.0127 (7)	0.0097 (6)	0.0028 (6)
C18	0.0165 (7)	0.0123 (7)	0.0143 (7)	0.0011 (5)	0.0057 (5)	0.0006 (5)
C19	0.0119 (6)	0.0123 (6)	0.0149 (7)	0.0008 (5)	0.0052 (5)	−0.0010 (5)
C20	0.0168 (7)	0.0151 (7)	0.0185 (7)	0.0014 (5)	0.0083 (6)	0.0016 (6)
C21	0.0176 (7)	0.0216 (8)	0.0133 (7)	0.0016 (6)	0.0061 (6)	0.0000 (6)
C22	0.0186 (7)	0.0171 (7)	0.0161 (7)	−0.0010 (6)	0.0075 (6)	−0.0038 (6)
C23	0.0123 (6)	0.0113 (6)	0.0157 (7)	−0.0001 (5)	0.0043 (5)	−0.0018 (5)
C24	0.0159 (7)	0.0138 (7)	0.0174 (7)	−0.0010 (5)	0.0072 (6)	−0.0013 (6)
C25	0.0153 (7)	0.0144 (7)	0.0275 (8)	−0.0020 (5)	0.0102 (6)	−0.0054 (6)
C26	0.0131 (7)	0.0176 (8)	0.0247 (8)	0.0008 (5)	0.0026 (6)	−0.0057 (6)
C27	0.0179 (7)	0.0218 (8)	0.0166 (7)	0.0044 (6)	0.0022 (6)	−0.0007 (6)
C28	0.0151 (7)	0.0203 (8)	0.0155 (7)	0.0027 (6)	0.0051 (6)	0.0004 (6)
C29	0.0189 (7)	0.0149 (7)	0.0289 (9)	−0.0015 (6)	0.0065 (6)	−0.0021 (7)

### Geometric parameters (Å, °)

**Table:**

Cl1—C4	1.7410 (18)	C13—H13A	0.9300
S1—C22	1.7163 (17)	C14—C15	1.391 (2)
S1—C19	1.7175 (16)	C14—H14A	0.9300
N1—C9	1.320 (2)	C15—H15A	0.9300
N1—C1	1.370 (2)	C16—C17	1.507 (2)
N2—C16	1.286 (2)	C17—C18	1.540 (2)
N2—N3	1.4048 (18)	C17—H17A	0.9700
N3—C23	1.4087 (19)	C17—H17B	0.9700
N3—C18	1.489 (2)	C18—C19	1.505 (2)
C1—C6	1.418 (2)	C18—H18A	0.9800
C1—C2	1.418 (2)	C19—C20	1.368 (2)
C2—C3	1.370 (3)	C20—C21	1.432 (2)
C2—H2A	0.9300	C20—H20A	0.9300
C3—C4	1.410 (2)	C21—C22	1.364 (2)
C3—H3A	0.9300	C21—H21A	0.9300
C4—C5	1.372 (2)	C22—H22A	0.9300
C5—C6	1.418 (2)	C23—C28	1.399 (2)
C5—H5A	0.9300	C23—C24	1.403 (2)
C6—C7	1.429 (2)	C24—C25	1.393 (2)
C7—C8	1.384 (2)	C24—H24A	0.9300
C7—C10	1.488 (2)	C25—C26	1.384 (3)
C8—C9	1.438 (2)	C25—H25A	0.9300
C8—C16	1.485 (2)	C26—C27	1.393 (2)
C9—C29	1.502 (2)	C26—H26A	0.9300
C10—C11	1.396 (2)	C27—C28	1.394 (2)
C10—C15	1.397 (2)	C27—H27A	0.9300
C11—C12	1.392 (2)	C28—H28A	0.9300
C11—H11A	0.9300	C29—H29A	0.9600
C12—C13	1.388 (2)	C29—H29B	0.9600
C12—H12A	0.9300	C29—H29C	0.9600
C13—C14	1.392 (2)		
			
C22—S1—C19	92.31 (8)	N2—C16—C8	121.76 (14)
C9—N1—C1	118.71 (14)	N2—C16—C17	114.33 (14)
C16—N2—N3	109.26 (13)	C8—C16—C17	123.90 (13)
N2—N3—C23	115.46 (12)	C16—C17—C18	102.24 (13)
N2—N3—C18	111.06 (12)	C16—C17—H17A	111.3
C23—N3—C18	120.22 (12)	C18—C17—H17A	111.3
N1—C1—C6	122.98 (14)	C16—C17—H17B	111.3
N1—C1—C2	117.65 (15)	C18—C17—H17B	111.3
C6—C1—C2	119.37 (15)	H17A—C17—H17B	109.2
C3—C2—C1	120.84 (16)	N3—C18—C19	112.48 (13)
C3—C2—H2A	119.6	N3—C18—C17	102.95 (12)
C1—C2—H2A	119.6	C19—C18—C17	112.01 (13)
C2—C3—C4	119.08 (15)	N3—C18—H18A	109.7
C2—C3—H3A	120.5	C19—C18—H18A	109.7
C4—C3—H3A	120.5	C17—C18—H18A	109.7
C5—C4—C3	122.16 (16)	C20—C19—C18	126.44 (14)
C5—C4—Cl1	119.42 (13)	C20—C19—S1	111.43 (12)
C3—C4—Cl1	118.41 (12)	C18—C19—S1	122.04 (11)
C4—C5—C6	119.31 (15)	C19—C20—C21	112.18 (15)
C4—C5—H5A	120.3	C19—C20—H20A	123.9
C6—C5—H5A	120.3	C21—C20—H20A	123.9
C1—C6—C5	119.20 (14)	C22—C21—C20	112.77 (14)
C1—C6—C7	117.69 (14)	C22—C21—H21A	123.6
C5—C6—C7	123.10 (14)	C20—C21—H21A	123.6
C8—C7—C6	118.57 (14)	C21—C22—S1	111.31 (12)
C8—C7—C10	121.26 (13)	C21—C22—H22A	124.3
C6—C7—C10	120.14 (14)	S1—C22—H22A	124.3
C7—C8—C9	119.51 (14)	C28—C23—C24	119.24 (14)
C7—C8—C16	119.99 (14)	C28—C23—N3	121.11 (13)
C9—C8—C16	120.44 (14)	C24—C23—N3	119.64 (14)
N1—C9—C8	122.51 (15)	C25—C24—C23	119.85 (15)
N1—C9—C29	116.62 (15)	C25—C24—H24A	120.1
C8—C9—C29	120.87 (14)	C23—C24—H24A	120.1
C11—C10—C15	119.21 (14)	C26—C25—C24	121.11 (15)
C11—C10—C7	120.38 (14)	C26—C25—H25A	119.4
C15—C10—C7	120.40 (13)	C24—C25—H25A	119.4
C12—C11—C10	120.37 (15)	C25—C26—C27	118.97 (15)
C12—C11—H11A	119.8	C25—C26—H26A	120.5
C10—C11—H11A	119.8	C27—C26—H26A	120.5
C13—C12—C11	120.23 (15)	C26—C27—C28	120.94 (16)
C13—C12—H12A	119.9	C26—C27—H27A	119.5
C11—C12—H12A	119.9	C28—C27—H27A	119.5
C12—C13—C14	119.69 (15)	C27—C28—C23	119.87 (15)
C12—C13—H13A	120.2	C27—C28—H28A	120.1
C14—C13—H13A	120.2	C23—C28—H28A	120.1
C15—C14—C13	120.31 (15)	C9—C29—H29A	109.5
C15—C14—H14A	119.8	C9—C29—H29B	109.5
C13—C14—H14A	119.8	H29A—C29—H29B	109.5
C14—C15—C10	120.20 (15)	C9—C29—H29C	109.5
C14—C15—H15A	119.9	H29A—C29—H29C	109.5
C10—C15—H15A	119.9	H29B—C29—H29C	109.5
			
C16—N2—N3—C23	−144.75 (14)	C13—C14—C15—C10	0.2 (2)
C16—N2—N3—C18	−3.41 (17)	C11—C10—C15—C14	0.1 (2)
C9—N1—C1—C6	−0.4 (2)	C7—C10—C15—C14	178.69 (15)
C9—N1—C1—C2	179.44 (14)	N3—N2—C16—C8	−177.80 (13)
N1—C1—C2—C3	−178.97 (15)	N3—N2—C16—C17	1.13 (19)
C6—C1—C2—C3	0.9 (2)	C7—C8—C16—N2	−120.77 (17)
C1—C2—C3—C4	0.9 (3)	C9—C8—C16—N2	61.8 (2)
C2—C3—C4—C5	−1.6 (3)	C7—C8—C16—C17	60.4 (2)
C2—C3—C4—Cl1	177.65 (13)	C9—C8—C16—C17	−117.06 (18)
C3—C4—C5—C6	0.4 (3)	N2—C16—C17—C18	1.44 (19)
Cl1—C4—C5—C6	−178.82 (12)	C8—C16—C17—C18	−179.66 (14)
N1—C1—C6—C5	177.80 (15)	N2—N3—C18—C19	124.85 (13)
C2—C1—C6—C5	−2.0 (2)	C23—N3—C18—C19	−95.91 (16)
N1—C1—C6—C7	−1.0 (2)	N2—N3—C18—C17	4.09 (16)
C2—C1—C6—C7	179.24 (14)	C23—N3—C18—C17	143.34 (14)
C4—C5—C6—C1	1.4 (2)	C16—C17—C18—N3	−3.15 (16)
C4—C5—C6—C7	−179.95 (15)	C16—C17—C18—C19	−124.23 (14)
C1—C6—C7—C8	2.0 (2)	N3—C18—C19—C20	−176.58 (14)
C5—C6—C7—C8	−176.68 (15)	C17—C18—C19—C20	−61.2 (2)
C1—C6—C7—C10	179.99 (14)	N3—C18—C19—S1	−0.30 (18)
C5—C6—C7—C10	1.3 (2)	C17—C18—C19—S1	115.10 (14)
C6—C7—C8—C9	−1.8 (2)	C22—S1—C19—C20	−0.18 (12)
C10—C7—C8—C9	−179.77 (14)	C22—S1—C19—C18	−176.97 (13)
C6—C7—C8—C16	−179.31 (14)	C18—C19—C20—C21	176.45 (14)
C10—C7—C8—C16	2.7 (2)	S1—C19—C20—C21	−0.17 (17)
C1—N1—C9—C8	0.6 (2)	C19—C20—C21—C22	0.6 (2)
C1—N1—C9—C29	179.45 (14)	C20—C21—C22—S1	−0.68 (18)
C7—C8—C9—N1	0.5 (2)	C19—S1—C22—C21	0.50 (13)
C16—C8—C9—N1	177.97 (14)	N2—N3—C23—C28	−13.4 (2)
C7—C8—C9—C29	−178.28 (15)	C18—N3—C23—C28	−151.01 (15)
C16—C8—C9—C29	−0.8 (2)	N2—N3—C23—C24	167.96 (14)
C8—C7—C10—C11	60.0 (2)	C18—N3—C23—C24	30.4 (2)
C6—C7—C10—C11	−117.87 (16)	C28—C23—C24—C25	1.9 (2)
C8—C7—C10—C15	−118.56 (17)	N3—C23—C24—C25	−179.51 (14)
C6—C7—C10—C15	63.5 (2)	C23—C24—C25—C26	−1.3 (2)
C15—C10—C11—C12	−0.2 (2)	C24—C25—C26—C27	0.1 (3)
C7—C10—C11—C12	−178.85 (14)	C25—C26—C27—C28	0.5 (3)
C10—C11—C12—C13	0.1 (2)	C26—C27—C28—C23	0.1 (3)
C11—C12—C13—C14	0.1 (3)	C24—C23—C28—C27	−1.3 (2)
C12—C13—C14—C15	−0.3 (3)	N3—C23—C28—C27	−179.88 (15)

### Hydrogen-bond geometry (Å, °)

**Table:**

*D*—H···*A*	*D*—H	H···*A*	*D*···*A*	*D*—H···*A*
C15—H15A···N1^i^	0.93	2.60	3.490 (2)	161
C3—H3A···Cg1^ii^	0.93	2.63	3.481 (2)	152
C12—H12A···Cg1^iii^	0.93	2.83	3.487 (2)	129
C17—H17B···Cg2	0.97	2.88	3.6916 (19)	142
C20—H20A···Cg3^iii^	0.93	2.89	3.7523 (18)	155
C21—H21A···Cg4^iv^	0.93	2.84	3.6084 (18)	141
C22—H22A···Cg3^v^	0.93	2.88	3.5750 (18)	132
C29—H29B···Cg5^vi^	0.96	2.89	3.694 (2)	142

Symmetry codes: (i) −*x*+1, *y*−1/2, −*z*+3/2; (ii) −*x*+1, *y*−1/2, −*z*−1/2; (iii) −*x*, *y*−1/2, −*z*−1/2;  (iv) *x*, −*y*−3/2, *z*−1/2; (v) −*x*, *y*+1/2, −*z*−1/2; (vi) −*x*+1, *y*+1/2, −*z*−1/2.
